# Integrative Multi-Omics Reveals Quality Markers and Metabolic Pathways Across Genotype and Ripening Gradients in High-Altitude *Malus*

**DOI:** 10.3390/foods14234039

**Published:** 2025-11-25

**Authors:** Huiqin Shi, Ting Guo, Chenlong Wei, Jie Tian, Xiaoqing Hou, Yi Li

**Affiliations:** 1 Qinghai Key Laboratory of Vegetable Genetics and Physiology, Academy of Agriculture and Forestry Sciences of Qinghai University, Xining 810016, China; 2 College of Agriculture and Animal Husbandry, Qinghai University, Xining 810016, China; 3 Key Laboratory of Food Nutrition and Safety, Ministry of Education, College of Food Science and Engineering, Tianjin University of Science & Technology, Tianjin 300457, China; lori_531@163.com; 4 Laboratory for Research and Utilization of Germplasm Resources in Qinghai-Tibet Plateau, Qinghai Academy of Agricultural and Forestry Sciences, Xining 810016, China

**Keywords:** *Malus*, multi-omics, organic acids, sugars, metabolic pathways

## Abstract

Apples from high-altitude orchards exhibit a distinctive sugar–acid balance, yet the varietal and maturity drivers remain unclear. A multi-omics survey was conducted to map sugars and acids and to resolve pathway control. Chromatography–mass spectrometry, RNA-seq, and principal component (PC) dimensionality reduction fitting analysis were integrated across *Malus prunifolia* and *M. asiatica* at 60 and 120 days after flowering (DAF). PC separated maturity on PC1 and species on PC2, and canonical correlation confirmed gene–metabolite couplings. Malate predominated but declined with ripening as sucrose rose; *M. prunifolia* at 60 DAF showed higher maltose/inositol, whereas *M. asiatica* at 120 DAF accumulated pentoses/xylitol. In *M. prunifolia* 60 vs. 120 DAF (Q60–Q120), enrichment targeted starch/sucrose plus pentose–glucuronate remodeling; in *M. asiatica* 60 vs. 120 DAF (S60–S120), fructose/mannose routes dominated with persistent wall-sugar signatures. Correlations linked succinate and pyruvate positively, and malate negatively, to hexoses, outlining an ALMT9/MDH-bridged acid–sugar switch. These maturation-anchored markers inform quality breeding and postharvest strategies.

## 1. Introduction

High-altitude environments on the northeastern QiFeau foster apple fruit with vivid peel coloration and distinctive sugar/acid balance, while strong solar radiation and large diurnal temperature ranges promote anthocyanin biosynthesis and favor soluble-solids accumulation [1]. Especially in the Ledu District (Haidong City, Qinghai Province, China), orchards lie at about 2125 m and 2543 sunshine hours annually, providing long-duration sunlight favorable for coloration and carbohydrate accumulation [2]. In addition, the fruit trees in this region possess unique ecological adaptability, stress resistance, and genetic diversity, making them invaluable resources indispensable in the development of modern agriculture. Within Chinese *Malus* germplasm, *Malus asiatica* and *M. prunifolia* (Willd.) Borkh. are long-cultivated northern taxa recorded in the Flora of China, where *M. prunifolia* is also widely used as a stress-tolerant rootstock [3]. The flowering periods of *M. asiatica* and *Malus prunifolia* trees are similar. In the Qinghai region, early April is the initial flowering period, mid-April is the peak flowering period, and late April and early May are the final flowering periods. There has been no significant change in the flowering period in the past three years. Functionally, overexpression of the *M. prunifolia* kinase MpSnRK2.10 confers enhanced drought tolerance in Arabidopsis and apple, and comparative physiology shows *M. prunifolia* exhibits stronger antioxidative capacity under drought than *M. hupehensis*, which traits aligning with high-altitude, water-limited production zones [4].

Beyond agro-climatic advantages, apples are selectively grown for nutrient density and sensory quality. Apples supply digestible carbohydrates (fructose, glucose, sucrose), pectin fiber, vitamin C, and diverse phytochemicals (phenolic acids, flavonoids, triterpenes) linked to antioxidant and cardiometabolic benefits [5]. Within Chinese crabapple germplasm, *M. asiatica* is particularly valued in China as a nutrient-rich fruit resource, as previous reports have noted its abundant vitamins, organic acids, and dietary fiber [6]. By contrast, *M. hupehensis* contains polyphenols, polysaccharides, amino acids, and volatiles with documented bioactivities, thereby supporting its reputation for high nutritional quality [6]. Across *Malus*, sugars and organic acids are primary quality drivers that shape sweetness-sourness, consumer liking, and processing performance [7]. In ripe apples, fructose is typically the principal sugar (with glucose and sucrose), sorbitol acts as a major translocated polyol, and malic acid predominates among organic acids (with cultivar-dependent citric/quinic contributions); concentrations vary with genotype and environment and discriminate cultivars and maturity stages [8].

For the ripening continuum, apple primary metabolism is reprogrammed in sequential stages [9]. In the early phase, developing fruit relies on imported sorbitol and sucrose, channeling carbon through glycolysis and the tricarboxylic-acid (TCA) cycle and maintaining relatively high organic-acid pools. During mid-development, carbon is preferentially allocated to transient starch deposition. Finally, in late ripening, starch is remobilized into soluble sugars (principally sucrose and fructose) while malic acid declines through dilution and catabolic loss, thereby lowering titratable acidity and increasing perceived sweetness [9]. These trajectories depend on both genotype and environment. Geography and cultivar jointly determine the sugar–acid endpoints: for example, many wild *Malus* accessions are dominated by glucose and fructose, whereas cultivated apples tend to accumulate higher levels of sucrose and fructose [10]. Moreover, altitude and the local thermal regime further modulate these final balances [7]. At the regulatory level, the vacuolar H^+^-pyrophosphatase MdVHP1-2 tracks rising sugar/acid ratios during ripening and, when overexpressed, elevates soluble sugars and shifts the sugar–acid balance in fruit [11].

These compositional axes (sugar/acid balance) therefore provide tractable biochemical markers for variety selection and ripening assessment, motivating a multi-omics focus on carbohydrate and organic-acid metabolism in *M. asiatica* and *M. hupehensis*. In this study, organic acid and sugar profiles of *Malus* fruits of different varieties (*M. asiatica* and *M. hupehensis*) and maturity (picked 60 and 120 days after flowering) were characterized, and transcriptome-wide gene-coexpression network analyses were applied to elucidate the mechanisms underlying quality formation.

## 2. Materials and Methods

### 2.1. Plant Materials

**Figure 1:**
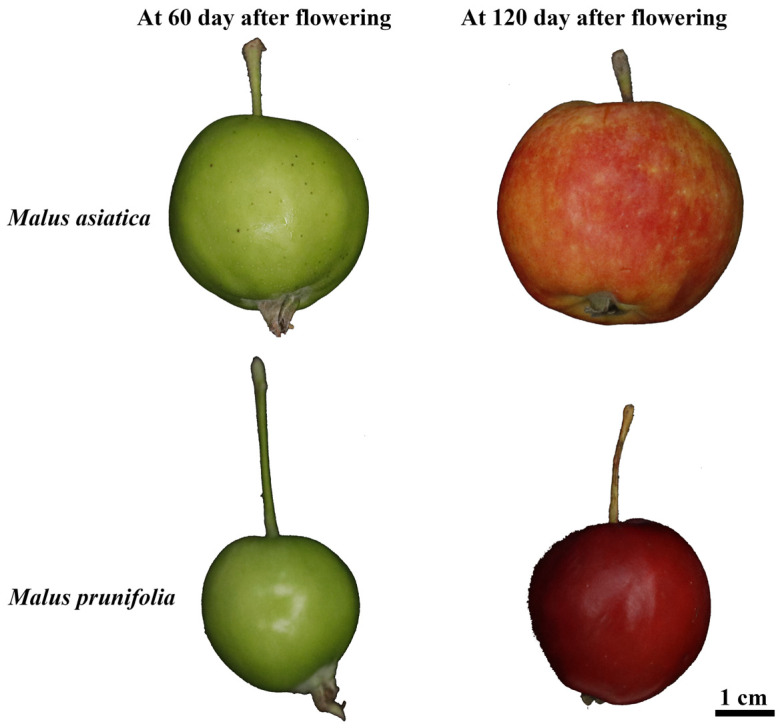
The fruits of *M. asiatica* and *M. prunifolia* at 60 days and 120 days after flowering, respectively.

*M. asiatica* and *M. prunifolia* are genus *Malus* native to China, which can be used as rootstocks (vigorous stock). The fruits of *M. asiatica* and *M. prunifolia* were collected from trees that are 30 years old, with soil consisting of clayey soil, and are naturally growing trees. Fruits of *M. asiatica* and *M. prunifolia* were harvested at two developmental stages (60 and 120 days after flowering, DAF) from the heritage variety conservation orchard in Ledu District (Haidong City, Qinghai Province, China; Altitude: 1942 m, N: 36.4746, E: 102.4479; Collection year: 2024). Four groups were defined: S60 and S120 (*M. asiatica* at 60 and 120 days after flowering) and Q60 and Q120 (*M. prunifolia* at 60 and 120 days after flowering) (Figure 1).

During sampling, four vigorous fruit trees were selected. Fruits were selected from the four directions of east, south, west, and north of each tree, respectively. Only intact, visibly healthy fruit free of pest or disease symptoms were selected. A total of 500 g of physiologically uniform fruits for each genotype and stage was taken.

For each genotype × developmental stage, we collected equatorial cortex tissue (edible flesh between peel and core), avoiding peel and seeds. After disinfection, tissues were pooled, thoroughly mixed in liquid nitrogen, and ground to a fine powder (MM 400, Retsch, Haan, Germany; 30 Hz, 1.5 min, with zirconia bead). Three independent biological replicates were prepared, each as an independently pooled and ground batch of fruits. From these homogenized powders, 0.05 g subsamples were used for metabolite extraction (Section 2.2 and Section 2.3) and 100 mg frozen powder was used for RNA extraction (Section 2.4), so that each analytical sample represents a composite of multiple fruits rather than a single individual.

### 2.2. Organic-Acid Components Assay

#### 2.2.1. Sample Pretreatment

Fruit sampling followed the design described in Section 2.1. A total of 0.05 g of powder was extracted with 500 µL of 70% (*v*/*v*) methanol/water in a 2 mL tube [12]. The mixture was vortexed for 3 min at 2500 rpm, then centrifuged at 12,000 rpm for 10 min at 4 °C. A 300 µL aliquot of the supernatant was transferred to a fresh tube and precipitated at −20 °C for 30 min, followed by a second centrifugation (12,000 rpm, 10 min, 4 °C). The final supernatant (200 µL) was filtered through a 0.22 µm PTFE membrane and placed in liquid chromatograph mass spectrometer (LC-MS) vials for analysis [13].

#### 2.2.2. Liquid Chromatography–Mass Spectrometry Conditions

Organic-acid analysis was performed on a UHPLC system (ExionLC™ AD, SCIEX, Framingham, MA, USA) coupled to a triple-quadrupole/linear ion trap mass spectrometer (QTRAP^®^ 6500+, SCIEX, Framingham, MA, USA) equipped with an electrospray ionization (ESI) source [14]. Chromatography used an ACQUITY HSS T3 column (Waters Corporation, Milford, MA, USA, 1.8 µm, 100 × 2.1 mm) at 40 °C. Mobile phases A (water with 0.05% formic acid) and B (acetonitrile with 0.05% formic acid) followed the gradient: 0–8 min, A 95 to 5%; 8–9.5 min, A 5%; 9.5–12 min, A 95% (re-equilibration), with flow 0.35 mL·min^−1^, injection 2 µL. MS conditions: ESI 550 °C, +5.5/−4.5 kV, CUR 35 psi, GS1/GS2 55/60 psi, collision gas “medium”. Data acquired in scheduled MRM (positive and negative); analyte-specific transitions and destocking potential/collision energy were optimized by standard infusion.

#### 2.2.3. Quantification of Organic-Acid Concentration

External calibration solutions at 0.01–10,000 ng·mL^−1^ were prepared (Table S1), and peak areas were plotted against concentrations to generate compound-specific calibration curves (linear regression with 1/x weighting; *R*^2^ ≥ 0.99). Sample concentrations (*c*, ng·mL^−1^) were obtained by substituting integrated peak areas into the relevant curve. Organic-acid content (ng·g^−1^ DW) was calculated as:

(1) $$Content=\frac{c\times v\;\times DF}{1000\times m}$$

where *v* is the extraction volume (µL; 500 µL unless otherwise noted), *DF* is any dilution factor applied prior to injection, and *m* is the weighed sample mass (g).

### 2.3. Sugars Components Assay

#### 2.3.1. Sample Pretreatment

Fruit sampling followed the design described in Section 2.1. A 0.05 g aliquot was extracted with 500 μL 70% (*v*/*v*) methanol/water, vortex-mixed 3 min, ultrasonicated 30 min, and centrifuged (12,000 rpm, 3 min, 4 °C) [12]. For gas chromatography–mass spectrometry (GC-MS; TMS-oxime) analysis, 50 μL supernatant was combined with 20 μL internal standard (1000 μg·mL^−1^), evaporated under N_2_, and lyophilized. The residue was methoximated with 100 μL methoxyamine hydrochloride in pyridine (15 mg·mL^−1^, 37 °C, 2 h), then silylated with 100 μL BSTFA (37 °C, 30 min). Samples were diluted appropriately before injection [15].

#### 2.3.2. Gas Chromatography–Mass Spectrometry Conditions

GC-MS system: 7890B GC coupled to a 5977B MSD (Agilent Technologies, Santa Clara, CA, USA) [16]. GC-MS system conditions: carrier gas, helium (1.0 mL·min^−1^); column: DB-5MS (30 m × 0.25 mm × 0.25 μm); oven, 160 °C (1 min), 6 °C·min^−1^ to 200 °C, 10 °C·min^−1^ to 270 °C, 20 °C·min^−1^ to 320 °C (hold 5.5 min); ionization, EI 70 eV; acquisition: SIM.

#### 2.3.3. Quantification of Sugar Concentration

Calibration standards (0.001–50 μg·mL^−1^) were prepared (Table S2). For GC-MS, Standard curves were constructed using analyte/internal-standard peak-area ratios versus concentration ratios. The concentration (mg·g^−1^ DW) in the processed sample (*c*, ng·mL^−1^) was obtained from the calibration; sugar content was calculated as:

(2) $$Content=\frac{c\times V_{1}\;\times V_{2}}{V_{3}\times m\;\times \;10^{6}}$$

where *V*_1_ is the final constant-volume after derivatization (μL), *V*_2_ is the extraction volume accounted for in processing (μL), *V*_3_ is the supernatant volume carried forward (μL), and m is the sample mass (g).

### 2.4. RNA Extraction, Detection, and Sequencing

Fruit sampling followed the design described in Section 2.1. Total RNA was extracted from frozen, pulverized fruit tissue (100 mg) using a CTAB-based protocol optimized for polysaccharide/phenolic-rich plants and dissolved in 50 µL DEPC-treated water with on-column DNase I digestion. RNA quantity was measured on a Qubit fluorometer (ThermoFisher Scientific, Waltham, MA, USA); purity was checked spectrophotometrically (A260/280 1.9–2.1; A260/230 > 2.0); integrity was assessed by capillary electrophoresis (Qsep400, reporting RNA Quality Number, RQN), and samples with RQN ≥ 7 were advanced. Poly(A) + mRNA was enriched with oligo(dT) beads and converted to strand-specific libraries (thermal fragmentation, end-repair/A-tailing, adapter ligation, limited-cycle PCR; 300 bp inserts), then sequenced by Metware Biotechnology (Wuhan, China) on a DNBSEQ platform using PE150 chemistry [17].

### 2.5. Bioinformatics Analysis

Raw paired-end reads were quality-filtered with fastp v0.23 (adapter removal; read discarded if ambiguous bases > 10% or if low-quality bases, Q ≤ 20, >50% of length); only clean reads were used downstream. Transcript abundance was quantified against a Malus reference transcript set using Salmon v1.10.1 (quasi-mapping; bias correction enabled). Gene-level counts were derived with tximport for statistical testing. Samples with biological replicates were assessed by principal component analysis (PCA)/hierarchical clustering to confirm within-group coherence, then differential expression between groups was computed with DESeq2 (Wald test; size-factor normalization; dispersion shrinkage). Significance required FDR (BH-adjusted *p*) < 0.05 and |log_2_FC| ≥ 1. Functional enrichment used clusterProfiler (R package, version 4.12.6) for GO Biological Process and KEGG over-representation (hypergeometric test; BH adjustment; gene-set size 10–500), with all expressed genes as background. Where indicated, gene co-expression modules were inferred by WGCNA (signed network; soft-threshold power chosen by scale-free topology) and correlated with sugar/acid traits [18].

### 2.6. Statistical Analyses

Experiments followed a completely randomized design with three independent replicates. Statistical analyses were performed using IBM SPSS Statistics 26 (Chicago, IL, USA). Means were compared by one-way ANOVA with Duncan’s multiple range test (*p* < 0.05); different letters indicate significant differences in bar charts. For correlation/heatmap analyses, Pearson’s correlation was used, and significance is shown as * *p* < 0.05, ** *p* < 0.01, and *** *p* < 0.001. All tests were applied to single-year datasets. Samples were performed in triplicate. Statistical analysis was performed using IBM SPSS Statistics 26 (Chicago, IL, USA). Samples were performed in triplicate, and significant analyses were performed by one-way analysis of variance (ANOVA) employing Duncan’s multiple range test. Raw mass spectrometry data were processed in Analyst software (v1.6.3, SCIEX, Framingham, MA, USA). Metabolite intensities were log_10_-transformed and unit-variance scaled (mean-centered and divided by the standard deviation). Canonical correlation analysis (CCA) quantified cross-set associations between the transcriptome (X) and metabolome (Y) by estimating paired canonical variates *U* = *a′X* and *V* = *b′Y* that maximize corr (*U*, *V*); successive pairs are orthogonal. Variable-variates correlations on the first two axes were visualized as correlation-circle plots to identify contributors (|*r*| ≥ 0.4). Associations about acid–sugar conversion were evaluated using Spearman’s rank correlation. Heat maps and hierarchical clustering were generated in R (v4.3) with ComplexHeatmap. Figures were rendered in R with default settings unless stated otherwise.

## 3. Results and Discussion

### 3.1. Differential Analysis of Dominant Organic Acids and Sugars in Malus Fruits

**Figure 2:**
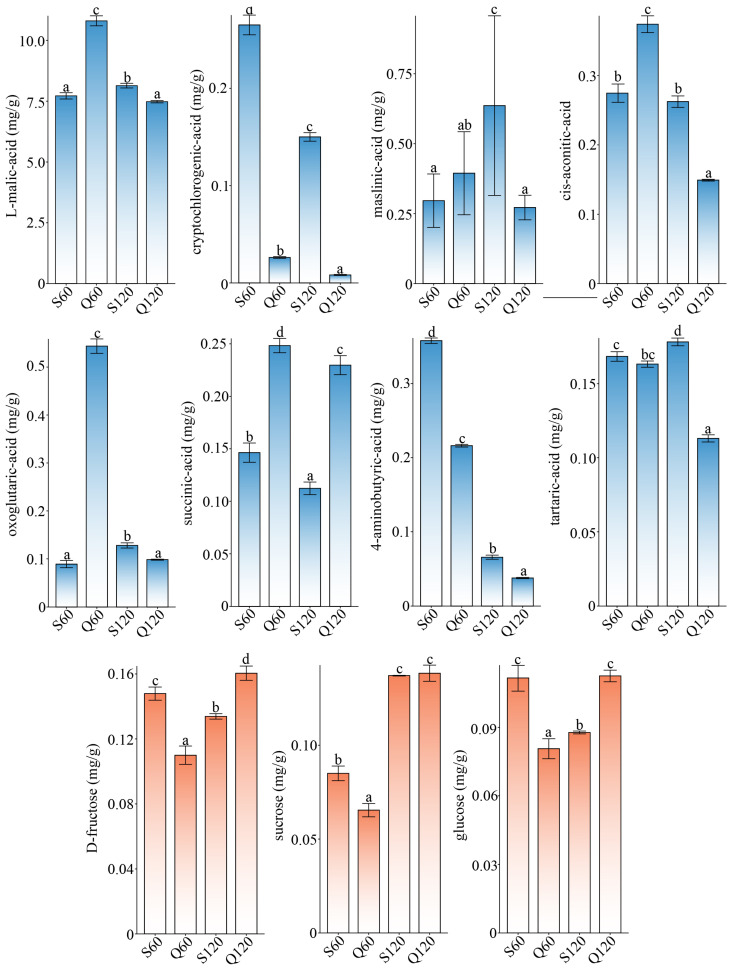
Differential analysis of organic acid and sugar components with concentration advantages (>0.1 mg/g). The blue and orange bar charts, respectively, represent the organic acid and sugar components, and the different letters a–d represent significant differences (*p* < 0.05).

Dominant organic acids and sugars (mg·g^−1^) were first compared (Figure 2, summarized data in Tables S3 and S4), which directly influence the overall quality of fruits. For organic acid, the pool was strongly malate-dominated, with the highest malate at Q60 and significant declines by Q120, a canonical maturation trend in apples where malate (often ≥70–90% of total acids) decreases via respiration and remobilization as ripening proceeds [9]. Concomitantly, several tricarboxylic-acid cycle (TCA) related acids showed stage- and species-specific shifts: cis-aconitate dropped most in Q120; oxoglutarate spiked at S120, indicating enhanced TCA anaplerosis; succinate peaked at Q60 then declined; tartaric acid trended downward to Q120; and γ-aminobutyric acid (GABA, 4-aminobutyrate) markedly rose at S120, consistent with activation of the GABA-shunt toward succinate and carbon/nitrogen buffering. Q60 therefore represents a high-malate, high-succinate early-ripening state, whereas by 120 DAF both Q120 and S120 show reduced malate and redistributed TCA intermediates, with S120 further characterized by strong GABA-shunt engagement, highlighting cultivar- and stage-dependent remodeling of organic acids. These observations align with established apple physiology: malate predominance and decline during maturation with dynamic redistribution of TCA/GABA intermediates [19].

For sugar, sucrose increased sharply at 120 DAF in both species (S120, Q120), while fructose rose along Q60, S60, S120, Q120, and glucose was lowest at Q60 and highest at Q120/S60, delineating a clear ripening-associated trajectory from low-sugar early fruit to high-sugar mature fruit. Mechanistically, this matches the Rosaceae carbon-partitioning model: sorbitol unloaded to the flesh is converted by sorbitol dehydrogenase into fructose and then glucose, while late-development upregulation of sucrose metabolism and starch hydrolysis elevates sucrose and hexoses [20]. Fructose and glucose typically predominate at harvest, with cultivar and environment modulating their ratios, a pattern concordant with the stronger hexose accumulation observed in *M. prunifolia* at maturity [21]. Q60 combines a high-malate/low-sugar profile, S60 already accumulates more hexoses, and by 120 DAF, both S120 and Q120 have shifted to low-acid, high-sugar states, with Q120 displaying pronounced hexose enrichment that matches its intrinsically hexose-rich sweetness.

### 3.2. Varietal and Maturity Effects on Common Organic-Acid Profiles in Malus Fruits

**Figure 3:**
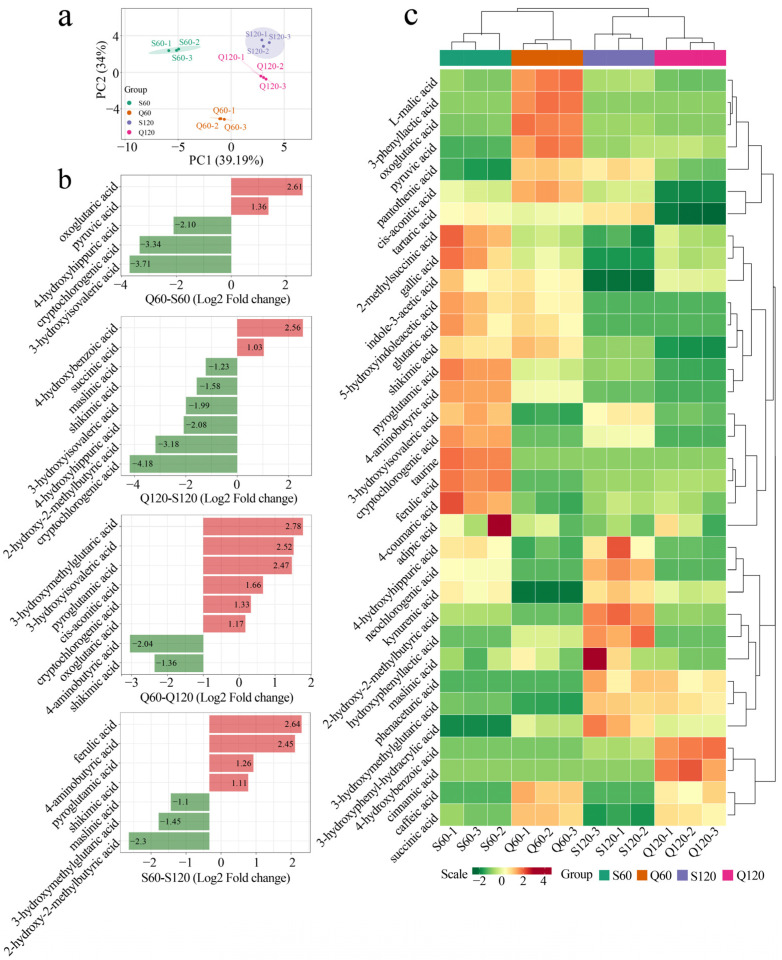
Comparison of organic acids from different varieties and maturity. (**a**) PCA of macroscopic organic-acid profiles. (**b**) Differences in organic acids between groups (fold change > 2). Bars represent log_2_-transformed fold changes calculated from the intensity ratios (Q60/S60, Q120/S120, Q60/Q120, and S60/S120). (**c**) Overall trends in organic-acid content (heatmap based on concentration).

Macroscopic profile and trends of organic acids were further analyzed (Table S3). In PCA (Figure 3a), PC1 (39.2%) cleanly separates 60 vs. 120 DAF across taxa, reflecting ripening-driven loss of L-malate (dominant acid) and concomitant remodeling of TCA intermediates, which, comparable to apple metabolomics, identify malate as a leading loading with stage-dependent redistributions of organic acids [22]. PC2 (34.0%) captures species differences consistent with increased GABA-shunt to succinate flux and differential malate handling; during development, organic acids are respired/remobilized and the GABA-shunt rises to feed succinate [23].

For comparison between Q60 and S60, *M. asiatica* at 60 DAF is enriched in oxoglutarate and pyruvate, consistent with stronger TCA entry/respiratory demand; in contrast, *M. prunifolia* shows higher cryptochlorogenic acid (4-CQA), 3-hydroxyisovaleric acid (3-HIVA), and 4-hydroxyhippuric acid, pointing to greater engagement of phenylpropanoid/benzoate pathways and branched-chain amino-acid (BCAA) catabolism at this stage. 4-CQA has been associated with aphid resistance in apples, and hydroxyhippuric acids are typical phenolic conjugates in plant/food metabolism [24]. For comparison between Q120 and S120, *M. asiatica* at 120 DAF accumulates more 4-hydroxybenzoic acid and succinate, whereas *M. prunifolia* is higher in shikimate, cryptochlorogenic acid, 3-HIVA, 2-hydroxy-2-methylbutyrate, and maslinic acid, where a profile suggesting species-specific routing toward benzoate/TCA vs. shikimate-derived and triterpenoid acids late in development; succinate gains at maturity/storage are widely observed as GABA-shunt/TCA flux increases [25]. For differences due to maturity (Q60–Q120 and S60–S120), within each species, malate falls from 60 to 120 DAF while succinate rise (Figure 2) as well as 4-aminobutyric acid and shikimic acid for Q120, and 3-hydroxymethylglutaric acid and 2-hydroxy-2-methylbutyric acid for S120, the canonical ripening pattern in apple; additional late increases in these components are consistent with enhanced TCA/BCAA turnover during maturation [26].

Heatmap clustering consolidates three themes: (i) a stage-dominant decline of the total acid pool led by malate, the major contributor to apple titratable acidity; (ii) network redistribution toward succinate/GABA with ripening; and (iii) a secondary varietal effect, with *M. prunifolia* retaining higher early acids while *M. asiatica* pivots earlier toward shikimate/phenolic-linked acids. These trajectories align with mechanistic genetics (variation at Ma1/ALMT9 (vacuolar malate transporter) underlies species/cultivar acidity differences) and with integrative studies showing that genetics and development jointly shape apple organic-acid endpoints [27].

### 3.3. Metabolic Pathways Mediating Organic-Acid Differences

Transcriptome enrichment coupled with CCA pinpointed the pathway logic behind organic-acid divergence across genotypes and ripening stages. The pronounced separations in Q60–S60 and Q60–Q120 were attributable to metabolic-pathway reprogramming (Table S5). By contrast, he differences between *M. asiatica* and *M. prunifolia* species (Q60–S60) and the maturation time of *M. asiatica* (S60–S120) were mainly reflected in progressive metabolite accumulation rather than distinct pathway switches. In line with the organic-acid profile, the strongest separations involved rebalancing within central carbon rather than wholesale pathway replacement: genotype effects were most evident at 60 DAF, whereas maturation primarily redirected flux from malate retention toward respiration-linked sinks [26].

**Figure 4:**
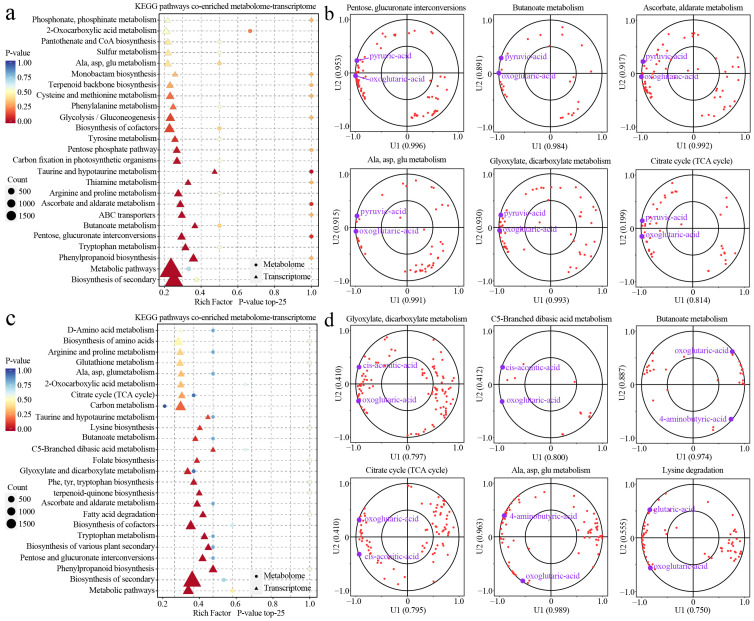
Metabolic-pathway profiles behind organic-acid differences. (**a**,**c**) represent the differentially expressed gene pathway enrichment in the Q60–S60 and Q60–Q120 groups, respectively. (**b**,**d**) represent canonical correlation analysis of differential metabolites and differential genes related to organic acids in the Q60–S60 and Q60–Q120 groups, respectively. Red dots represent individual genes (transcripts) in the corresponding KEGG pathway, projected onto the first two latent variables (U1 and U2) of the integrated metabolome–transcriptome model.

For Q60–S60 differential pathways (Figure 4a,b), early species divergence was anchored in 2-oxocarboxylic acid metabolism, glycolysis/gluconeogenesis, and Ala/Asp/Glu metabolism, with supporting signals from TCA, glyoxylate-dicarboxylate, butanoate (GABA), and transport terms. In the CCA space, pyruvate and 2-oxoglutarate carried the leading canonical loadings, indicating that *M. prunifolia* and *M. asiatica* allocate carbon differently at the pyruvate to acetyl-CoA entry and α-ketoglutarate anaplerosis/transamination nodes. Such positioning would modulate downstream malate/citrate pools via malate dehydrogenase, citrate synthase, and isocitrate dehydrogenase balance, while transporter activity provides a plausible lever on malate partitioning. This hub-centric view explains the stronger genotype separation at 60 DAF reported in the organic-acid profile, without invoking distinct pathway “on/off” states [28].

For Q60–Q120 differential pathways (Figure 4b,d), ripening in *M. prunifolia* broadened the reprogramming toward TCA and glyoxylate-dicarboxylate remodeling, with coordinated up-weighting of butanoate (GABA) metabolism and amino-acid catabolism (e.g., lysine to glutarate, C5-branched dibasic acids). The CCA projections of cis-aconitate, 2-oxoglutarate, GABA, and glutarate mirror these enrichments and indicate an operational route (glutamate decarboxylase, GABA transaminase, succinic semialdehyde dehydrogenase, succinate) that injects carbon into the TCA while supporting redox/pH homeostasis. Functionally, this accounts for the shift from vacuolar malate storage to respiratory utilization, thereby validating the malate (downregulation)/succinate (upregulation) maturation pattern and the PC1 separation without restating metabolite tables [29].

### 3.4. Varietal and Maturity Effects on Common Sugar Profiles in Malus Fruits

**Figure 5:**
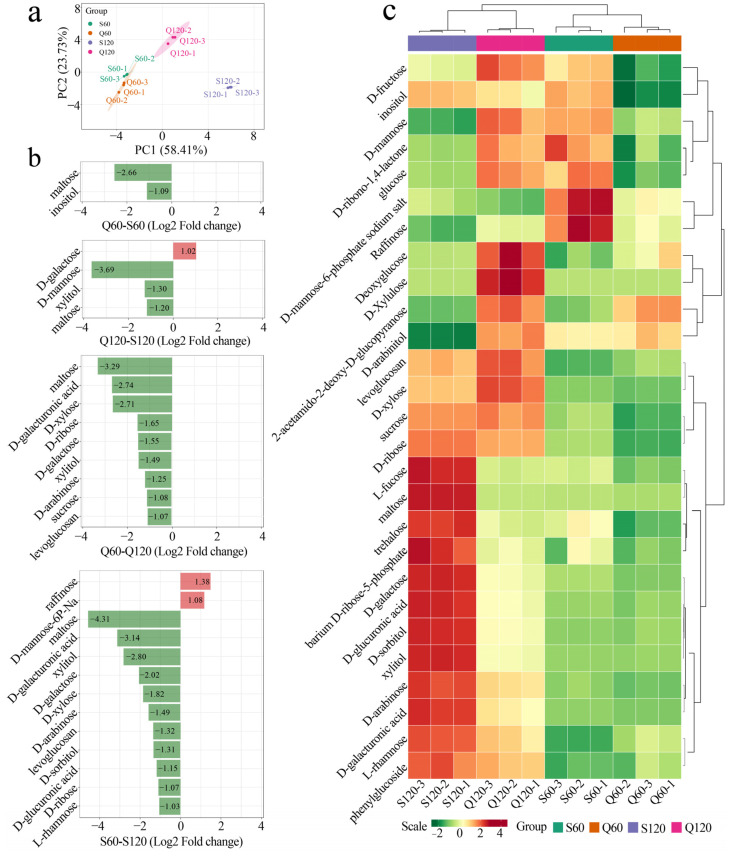
Comparison of sugar from different varieties and maturity. (**a**) PCA of macroscopic sugar profiles. (**b**) Differences in organic acids between groups (fold change > 2). Bars represent log_2_-transformed fold changes calculated from the intensity ratios (Q60/S60, Q120/S120, Q60/Q120, and S60/S120). (**c**) Overall trends in sugar content (heatmap based on concentration).

Macroscopic profile and trends of sugars were further analyzed (Table S4). In PCA (Figure 5a), both *M. asiatica* and *M. prunifolia* shift rightward from 60 to 120 DAF (signifying global sugar accrual) while *M. asiatica* and *M. prunifolia* comparison separate along with PC2. This mirrors the acid-side PCA (PC1 = maturity; PC2 = species), consistent with a concerted transition from a malate-rich early developmental state at 60 DAF toward a sugar-rich mature state by 120 DAF. Mechanistically, vacuolar malate partitioning via Aluminum-activated Malate Transporter 9/malic acid locus 1 (ALMT9/Ma1) and cytosolic malate dehydrogenase (which has been reported to influence sucrose synthesis and supply carbon to respiration) have been proposed in previous studies to link organic-acid metabolism, sucrose accumulation and respiratory carbon use, and therefore provide a physiologically plausible bridge between the acid and sugar spaces [28].

At 60 DAF (Q60–S60, Figure 5b), only maltose and inositol clear the threshold, and both are higher in Q60, indicating an early transient starch-to-soluble-sugar pulse (maltose) and inositol flux into wall precursors (UDP-GlcA) that support cell-wall construction, which processes known to rise during pre-ripening sink establishment [30]. By 120 DAF (Q120–S120), D-galactose, D-xylose, and xylitol are relatively lower in *M. asiatica*, pointing to stronger cell-wall remodeling/hemicellulose-pectin turnover in M. asiatica late in ripening. Within genotypes, maturation follows complementary logics: in Q60–Q120 most sugars rise (negative bars), with D-galacturonic acid increasing and maltose declining, which hallmarks of pectin de-esterification/depolymerization coupled to starch hydrolysis as *M. prunifolia* pivots to storage sugars; in S60–S120 the pattern is similarly upward overall but retains early D-mannose-6-phosphate/raffinose buffering, consistent with a more hexose-flexible trajectory in *M. asiatica* [31]. Mechanistically, these contrasts align with carbohydrate supply and sink programming: sorbitol to fructose provisioning in apple sinks, and late sucrose-phosphate synthase/sucrose synthase up-weighting that drives sucrose accrual, operate alongside the GABA-shunt that feeds succinate to respiration, which together explain why *M. asiatica* trends toward a sucrose-rich end-state while *M. prunifolia* retains wall/hexose signatures at 120 DAF [20].

The heatmap clusters primarily by maturity (120 DAF columns warmer across many sugars) and secondarily by species, revealing: (i) a pan-maturity rise in neutral sugars; and (ii) a wall-associated sub-cluster (e.g., galacturonic acid/pentoses) that increases with ripening and more strongly marks S at 120 DAF. This pattern dovetails with the acid-side evidence: ALMT9/Ma1-dependent easing of vacuolar malate pressure and malate dehydrogenase-mediated cytosolic redox favor sugar deposition, while cell-wall remodeling supplies galacturonic/pentose derivatives that co-vary with softening [28].

### 3.5. Metabolic Pathways Mediating Sugar Differences

**Figure 6:**
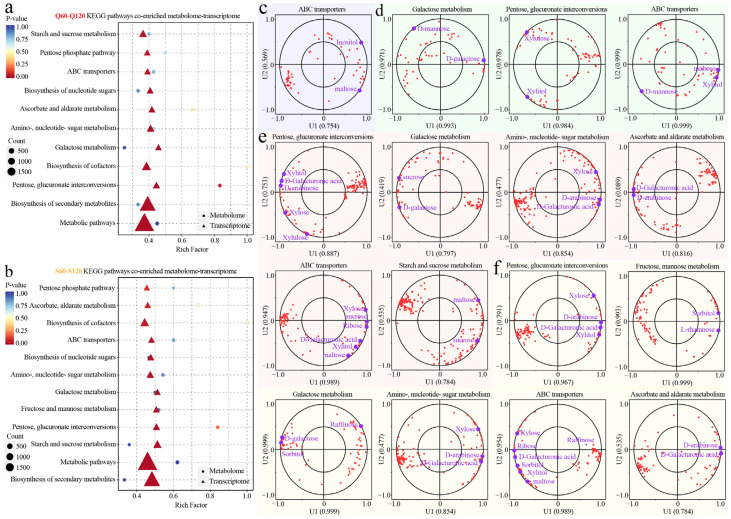
Metabolic-pathway profiles behind sugar differences. (**a**,**b**) represent the differentially expressed gene pathway enrichment in the Q60–Q120 and S60-S120 groups, respectively. (**c**–**f**) represent canonical correlation analysis of differential metabolites and differential genes related to sugars in Q60–S60 (purple background), Q120–S120 (green background), Q60–Q120 (red background), and S60–S120 (yellow background) groups, respectively. Red dots represent individual genes (transcripts) in the corresponding KEGG pathway, projected onto the first two latent variables (U1 and U2) of the integrated metabolome–transcriptome model.

CCA coupling between differential genes and sugars is strongest and broadest in the maturation pairs (Q60–Q120 and S60–S120, Table S6) across starch/sucrose, pentose–glucuronate, galactose, fructose/mannose, amino-/nucleotide-sugar, ascorbate/aldarate, nucleotide-sugar biosynthesis, and ABC transporters; inter-species pairs (Q60–S60, Q120–S120) show narrower coupling (Table S6, Figure 6c,d). Hence, Figure 6 centers on maturation pathways, with species biases read from the CCA panels.

For Q60–Q120 (Figure 6a), transcriptome–metabolome co-enrichment highlights starch/sucrose metabolism together with pentose–glucuronate and galactose modules, mapping a shift from starch-maltose toward soluble sugars alongside pectin remodeling (uronic acids, pentoses). The CCA plots align (Figure 6e): maltose vectors recede, while D-galacturonic acid/arabinose/xylose loadings strengthen, consistent with late-stage sucrose-phosphate synthase/sucrose synthase up-weighting that drives sucrose build-up and with wall de-esterification/depolymerization supplying uronates and pentoses [30]. The acid–sugar bridge operates here: cytosolic malate dehydrogenase elevates sucrose by tuning malate/redox status, while the ALMT9/Ma1 “acid valve” facilitates the switch from malate storage to sugar deposition.

For S60–S120 (Figure 6b), in *M. asiatica*, enrichment spans fructose/mannose with pentose–glucuronate and galactose routes; the CCA (Figure 6f) emphasizes vectors for sorbitol, L-rhamnose, xylose/xylitol, indicating a maturation program that increases soluble sugars yet retains hexose-biased flexibility with pronounced wall-sugar signatures. This matches apple’s sorbitol to fructose provisioning in sinks via SDH, which sustains hexose pools distinct from *M. prunifolia*’s more sucrose-storage-oriented trajectory [20]. Again, in line with organic-acid metabolism, ALMT9/Ma1-mediated relief of vacuolar malate pressure and malate dehydrogenase-coupled redox favor sucrose synthesis, while wall remodeling contributes galacturonic acid and pentoses captured by the CCA loadings [28].

### 3.6. Correlation Analysis of Key Organic Acids and Sugar Interconversions

**Figure 7:**
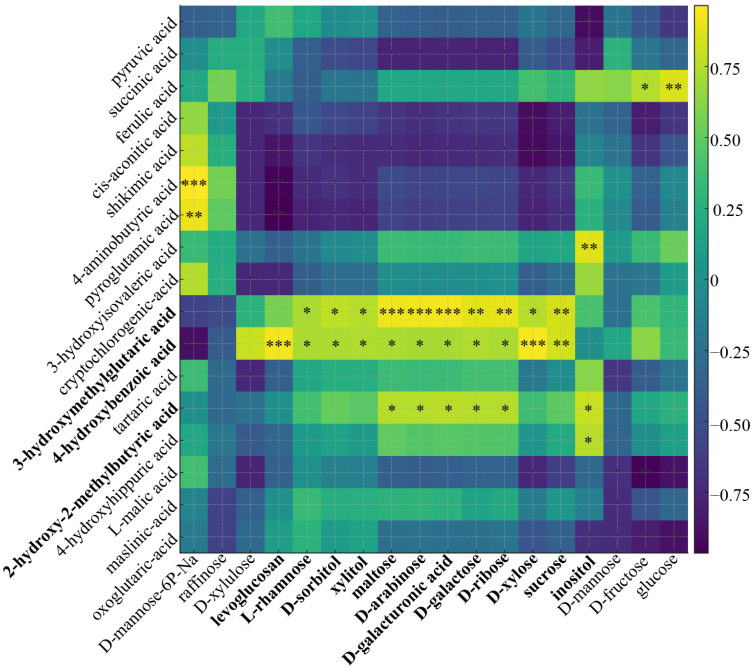
Conversion correlation analysis of key organic acids and sugars. The importance of the components was screened from Table S7. Bolded components indicate those that are significantly correlated with multiple metabolites, where * *p* < 0.05, ** *p* < 0.01, and *** *p* < 0.001.

The correlation heatmap (Figure 7) cross-links key organic acids (rows) with sugars and sugar derivatives (columns). Three coherent correlation blocks emerge that dovetail with the previous analyses: (i) a TCA/GABA block (pyruvate, succinate, cis-aconitate, GABA) that is positively associated with higher sugar levels, indicating coordinated changes between central organic-acid metabolism and sugar accrual; (ii) a cell-wall/uronate block (signals with D-galacturonic acid, pentoses and xylitol on the sugar side); and (iii) a phenylpropanoid/branched-chain acid block that co-varies with broad neutral-sugar lifting. These correlation blocks reflect coordinated shifts between central organic-acid metabolism and sugar interconversion during ripening.

For TCA/GABA-sugar coupling, rows for succinate and pyruvate show broad positive links to glucose/fructose (rightmost columns), whereas malate shows the opposite (predominantly negative) pattern, which precisely mirrors the maturation shift from malate to sugar. Two levers connect the acid and sugar spaces: (1) the ALMT9/Ma1 “acid valve” on the tonoplast, whose functional state controls vacuolar malate storage and thereby the cytosolic context for sugar deposition, and (2) cytosolic malate dehydrogenase, which raises sucrose by tuning malate/redox status in the cytosol [26,28]. The GABA-shunt furnishes succinate to the TCA cycle, sustaining respiration as sugars accumulate, consistent with the positive succinate-sugar correlations and our pathway results [32].

For cell-wall/uronate-sugar coupling, significant positive associations appear between acids that report cell-wall remodeling and sugar columns annotated as D-galacturonic acid, pentoses (xylose/arabinose), and xylitol. This pattern is consistent with pectin de-esterification/depolymerization during ripening, which liberates uronates and pentoses while softening the wall; the same carbohydrate signatures were enriched in glucose metabolism profiles for late stages (especially in *M. asiatica*) [33]. The KEGG pentose and glucuronate interconversions map provides the biochemical routes by which these wall sugars feed central carbon.

Extending the acid–sugar coupling described above, Figure 6 pinpoints a third, supply-side node centered on the myo-inositol/hexose-phosphate pool: inositol shows positive associations with wall-derived sugars (e.g., D-galacturonic acid, pentoses, xylitol), consistent with the myo-inositol-D-glucuronic acid-UDP-GlcA route that furnishes nucleotide-sugar precursors for pectin/hemicellulose backbones [34]. UDP-GlcA indeed serves as a common precursor for arabinose, xylose, and galacturonic acid residues of the primary cell wall, matching the “wall-sugar” fingerprint observed at early stages [35]. In parallel, D-mannose-6-phosphate correlates negatively with many soluble sugars, echoing a decline of early hexose-phosphate/RFO buffering as ripening advances, while the sorbitol-fructose conversion that typifies apple sinks sustains hexose influx into the soluble-sugar pool [36]. The directionality of acid–sugar correlations (malate negative; pyruvate/succinate positive) is mechanistically coherent with an ALMT9/Ma1-gated relief of vacuolar malate storage that opens the cytosolic window for sugar deposition and with cytosolic malate dehydrogenase control of malate/redox status that promotes sucrose synthesis [26,28]. Finally, the GABA-shunt supplies succinate to the TCA cycle to sustain respiration during sugar build-up, explaining why succinate tracks with neutral sugars across maturity and genotypes [37].

## 4. Conclusions

Using multi-omics and multivariate modeling, we establish a maturity-dominated axis of variation shared by acids and sugars and resolve genotype-specific carbon routing. Malate predominates yet decreases from 60 to 120 DAF as sucrose and hexoses increase; *M. prunifolia* follows a starch-sucrose plus wall-remodeling program, whereas *M. asiatica* retains hexose-biased and wall-sugar signatures. Correlation structure identifies an ALMT9/MDH-mediated acid–sugar switch, a GABA-succinate respiratory support, and an inositol-UDP-GlcA node linking wall metabolism to sugar status. Limitations include two time points and two taxes. Extending temporal resolution, adding targeted flux, and transporter kinetics will refine causal control points.

## Data Availability

The data underlying this article are available in the article and in its online Supplementary Material.

## Supplementary Materials

The following supporting information can be downloaded at: https://www.mdpi.com/article/10.3390/foods14234039/s1, Table S1: Organic acid standard curve information, Table S2: Sugar standard curve information, Table S3: Organic acid concentration (ng/g), Table S4: Sugar concentration (mg/g), Table S5: Canonical correlation analysis of differential metabolites and differential genes related to organic acids, Table S6: Canonical correlation analysis of differential metabolites and differential genes related to sugar, Table S7: Screening of key components for acid-sugar conversion analysis.
