# Supplementary material for: Integrative Multi-Omics Reveals Quality Markers and Metabolic Pathways Across Genotype and Ripening Gradients in High-Altitude Malus
Source: Foods. 2025 Nov 25;14(23):4039. doi: 10.3390/foods14234039 (PMC12691744; doi:10.3390/foods14234039)
Supplement: Supplementary file 1 [file foods-14-04039-s001.zip › foods-3993017-supplementary.pdf]

Integrative Multi-Omics Reveals Quality Markers and Metabolic Pathways Across Varietal and Ripening Gradients in High-Altitude *Malus*

Huiqin Shi [1,2,†], Ting Guo [3,†], Chenlong Wei [1,4], Jie Tian [1,4], Xiaoqing Hou [1,4] and Yi Li [1,4,*]

[1] Qinghai Key Laboratory of Vegetable Genetics and Physiology, Academy of Agriculture and Forestry Sciences of Qinghai University, Xining, 810016, China

[2] College of Agriculture and Animal Husbandry, Qinghai University, Xining 810016, China

[3] Key Laboratory of Food Nutrition and Safety, Ministry of Education, College of Food Science and Engineering, Tianjin University of Science & Technology, Tianjin 300457, China

[4] Laboratory for Research and Utilization of Germplasm Resources in Qinghai–Tibet Plateau, Qinghai Academy of Agricultural and Forestry Sciences, Xining 810016, China

[*] Corresponding authors, Email: Yi Li: 1994990028@qhu.edu.cn

[†] These authors contributed equally to this work

Table S1 Organic acid standard curve information

Index	RI	Equation	*R*	Weighting	LLOQ	ULOQ
4-aminobutyric-acid	0.73	y = 26128.82898 x + 1504.95995	0.99959	1/x	2	5000
taurine	0.76	y = 3.56959e4 x + 1121.77561	0.99896	1/x	0.5	2000
shikimic-acid	0.85	y = 3312.83657 x + 2492.02388	0.99499	1/x	2	2000
tartaric-acid	0.85	y = 1232.40571 x - 7.54212e5	0.99054	1/x	500	10000
L-malic-acid	0.86	y = 3920.65021 x + 5977.17160	0.99394	1/x	20	5000
pyruvic-acid	0.96	y = 1199.16125 x + 5.62407e4	0.99418	1/x	200	10000
lactic-acid	1.15	y = 960.27429 x + 1.65115e5	0.99219	1/x	20	2000
oxoglutaric-acid	1.15	y = 945.41826 x + 3.28726e4	0.99579	1/x	500	10000
pyroglutamic-acid	1.16	y = 2.28509e5 x + 2.12303e5	0.99455	1/x	0.5	500
maleic-acid	1.19	y = 12212.66881 x + 4.60208e4	0.99545	1/x	50	10000
fumaric-acid	1.29	y = 4424.58541 x + 27186.36225	0.99428	1/x	200	5000
cis-aconitic-acid	1.3	y = 4654.05575 x - 2.67228e6	0.99492	1/x	500	10000
succinic-acid	1.37	y = 14573.83622 x + 6.18144e4	0.99643	1/x	2	5000
3-D-hydroxybutyric-acid	1.41	y = 7836.23413 x + 9.56490e4	0.9962	1/x	50	2000
trans-aconitic-acid	1.44	y = 4906.98879 x - 4.05320e5	0.9936	1/x	200	10000
3-hydroxymethylglutaric-acid	1.5	y = 8020.32284 x + 605.59932	0.999	1/x	5	2000
methylmalonic-acid	1.54	y = 11750.87544 x - 29105.13567	0.9955	1/x	10	10000
crtraconic-acid	1.81	y = 3839.74232 x + 808.93285	0.99516	1/x	50	10000
gallic-acid	1.86	y = 2.35570e5 x + 5187.95580	0.99921	1/x	0.2	2000
glutaric-acid	2	y = 2848.93985 x + 22476.43201	0.99822	1/x	200	10000
levulinic-acid	2.03	y = 1939.93621 x + 1.57115e5	0.99108	1/x	200	10000
kynurenine	2.11	y = 5950.56990 x + 21679.67261	0.99939	1/x	20	5000
3-hydroxyisovaleric-acid	2.16	y = 3999.96945 x + 4372.17040	0.99975	1/x	5	2000
5-hydroxymethyl-2-furoic-acid	2.22	y = 11132.76810 x + 4716.38040	0.99779	1/x	2	2000
pantothenic-acid	2.35	y = 13136.18690 x + 1682.27597	0.99828	1/x	1	10000
2-methylsuccinic-acid	2.38	y = 13869.81524 x + 5.77495e4	0.99749	1/x	50	2000
ethylmalonic-acid	2.47	y = 6226.56885 x - 1.05950e5	0.99903	1/x	200	10000
2-hydroxy-2-methylbutyric-acid	2.54	y = 7870.69025 x + 2253.26225	0.99894	1/x	2	2000
4-hydroxyhippuric-acid	2.55	y = 7.66649e4 x + 1168.11568	0.99935	1/x	2	2000
neochlorogenic-acid	2.63	y = 7.90562e4 x - 881.32910	0.99559	1/x	0.5	5000
adipic-acid	2.66	y = 6079.86206 x + 23872.50586	0.99743	1/x	2	10000
2-hydroxyisovaleric-acid	2.75	y = 19182.24229 x + 895.20578	0.99824	1/x	1	1000
3-hydroxyhippuric-acid	2.76	y = 1.98072e5 x + 517.95840	0.99752	1/x	0.5	2000
3,4-dihydroxyphenylacetic-acid	2.78	y = 167.33777 x + 12082.45253	0.99444	1/x	100	2000
hydroxyphenyllactic-acid	2.8	y = 22249.10952 x + 10385.13446	0.99595	1/x	1	2000
kynurenic-acid	2.88	y = 3.92815e5 x + 6520.89051	0.99721	1/x	0.5	1000
3-Hydroxyphenyl-hydracrylic-acid	2.88	y = 28082.00050 x + 1707.42342	0.99814	1/x	0.5	2000
cryptochlorogenic-acid	3	y = 22156.77365 x - 6867.64836	0.99946	1/x	2	10000
5-hydroxyindoleacetic-acid	3.15	y = 1.14610e5 x + 32.56256	0.99961	1/x	0.5	2000
4-hydroxybenzoic-acid	3.15	y = 5.88130e4 x + 4.28764e4	0.99712	1/x	2	2000
3-methyladipic-acid	3.2	y = 1.13279e5 x + 8.18571e4	0.999	1/x	5	2000
4-hydroxyphenylacetic-acid	3.25	y = 6342.87368 x + 1.08552e5	0.99586	1/x	50	2000
caffeic-acid	3.3	y = 8.03749e4 x + 20921.77276	0.99854	1/x	5	5000
hippuric-acid	3.31	y = 21486.88832 x + 9223.79801	0.99799	1/x	2	2000

homovanillic-acid	3.43	y = 1599.20816 x + 4.84278e4	0.99612	1/x	200	10000
3-hydroxyphenylacetic-acid	3.46	y = 1000.24134 x + 4.09681e4	0.99761	1/x	100	2000
phenaceturic-acid	3.58	y = 1.10227e5 x + 1405.19954	0.99838	1/x	1	2000
2-hydroxyphenylacetic-acid	3.71	y = 6.54327e4 x + 4.94533e4	0.99563	1/x	5	2000
suberic-acid	3.75	y = 3.28524e4 x + 3.81516e5	0.99901	1/x	1	2000
4-coumaric-acid	3.79	y = 9.00399e4 x + 5071.81043	0.99879	1/x	2	2000
3-phenyllactic-acid	3.92	y = 7454.59212 x + 2706.11965	0.99882	1/x	5	10000
aminobenzoic-acid	3.93	y = 4.57934e5 x - 1750.89401	0.99804	1/x	0.5	1000
ferulic-acid	3.96	y = 20971.53054 x + 182.29375	0.99707	1/x	1	10000
indolelactic-acid	4.06	y = 3.79753e4 x + 2351.79048	0.99851	1/x	1	10000
azelaic-acid	4.27	y = 5.79158e4 x + 1.57474e6	0.99945	1/x	2	5000
benzoic-acid	4.44	y = 1723.77100 x + 22876.35567	0.99854	1/x	20	10000
indole-3-acetic-acid	4.52	y = 1.45225e6 x + 7652.83921	0.99703	1/x	0.05	200
salicylic-acid	4.6	y = 1.95808e5 x + 2.58577e5	0.99633	1/x	1	2000
sebacic-acid	4.78	y = 1.52293e5 x + 3.87192e6	0.99729	1/x	2	2000
2-indolecarboxylic-acid	5.01	y = 3.44629e4 x + 22636.28657	0.99667	1/x	5	5000
benzenepropanoic-acid	5.11	y = 470.24102 x - 4462.46330	0.99571	1/x	50	5000
cinnamic-acid	5.15	y = 7.02783e4 x + 11516.64276	0.99699	1/x	1	2000
maslinic-acid	8.41	y = 34.37950 x + 8874.57208	0.99473	1/x	50	2000
carnosic-acid	8.47	y = 73.46953 x - 329.31751	0.9989	1/x	20	10000
oleanic-acid	N/A	y = 844.64235 x + 86.36901	0.99926	1/x	50	10000

1) Index is the substance name
2) Equation is the linear equation
3) r is the correlation coefficient
4) Weighting is the weight
5) LLOQ (ng/mL) and ULOQ (ng/mL) are the lower limit of quantification and upper limit of quantification, respectively. Accurate quantification is possible within these limits.

Table S2 Sugar standard curve information

Index	RT	Equation	R2	Weighting	LLOQ	ULOQ
2-Deo-ribose	4.355	y = 0.269429 x - 1.802509E-004	0.994579	1/x	0.029	5
Xylose	5.247	y = 0.576670 x - 4.255107E-004	0.995123	1/x	0.009	5
D-Ara	5.334	y = 0.671955 x - 3.014585E-004	0.992397	1/x	0.014	5
Ribose	5.488	y = 0.931749 x + 6.433354E-004	0.998735	1/x	0.021	5
Xylulose	5.489	y = 1.119106 x + 0.004141	0.99632	1/x	0.021	5
Ribono-1-4-lactone	5.635	y = 0.224422 x - 8.599778E-005	0.999233	1/x	0.1	5
Xylitol	5.875	y = 1.227279 x - 2.180117E-004	0.999295	1/x	0.01	5
Lev	5.939	y = 0.208386 x - 7.536778E-005	0.997221	1/x	0.021	5
Rha	6.054	y = 0.581206 x - 3.476961E-004	0.996366	1/x	0.048	5
Arabinitol	6.055	y = 1.154880 x + 2.883221E-004	0.998974	1/x	0.04	5
Fuc	6.347	y = 0.061705 x - 1.809674E-005	0.998011	1/x	0.025	5
Deo	7.066	y = 0.179850 x - 9.190721E-005	0.992	1/x	0.032	5
1-5-Anh	7.931	y = 0.465095 x - 1.622783E-004	0.99249	1/x	0.027	5
Met	7.932	y = 0.659706 x - 6.114399E-004	0.992951	1/x	0.022	5
Fru	8.159	y = 0.567611 x - 0.007012	0.99724	1/x	0.006	50
Man	8.346	y = 0.327406 x - 1.457227E-004	0.993916	1/x	0.008	5
Gal	8.413	y = 0.244067 x - 2.085990E-004	0.992523	1/x	0.011	5
Glu	8.498	y = 0.353226 x - 0.003176	0.99419	1/x	0.011	50
Sorbitol	9.006	y = 1.841360 x - 2.318238E-004	0.992988	1/x	0.007	50
Glucuronic-A	9.007	y = 0.222641 x - 4.046254E-005	0.992702	1/x	0.009	5
Gal-A	9.108	y = 0.135689 x - 2.902203E-005	0.998353	1/x	0.012	5
2-Ace-2-Deo-D-Glucosamine	10.756	y = 0.027389 x - 2.009664E-005	0.993453	1/x	0.028	5
Inositol	10.865	y = 0.629855 x - 4.009578E-004	0.991626	1/x	0.013	25
Ribose-5-pho-Ba	11.082	y = 0.006295 x - 5.965607E-006	0.995889	1/x	0.074	5
Man-6-pho	13.083	y = 0.001677 x - 1.388285E-006	0.993038	1/x	0.023	5
Phe	13.14	y = 0.225412 x - 2.494822E-006	0.999476	1/x	0.004	5
Suc	15.935	y = 0.770994 x - 0.017173	0.990068	1/x	0.007	50
Lac	16.385	y = 0.039936 x - 3.135933E-005	0.997202	1/x	0.019	5
Cel	16.513	y = 0.091875 x - 2.245096E-005	0.995483	1/x	0.015	25
Mal	16.826	y = 0.082982 x - 1.073294E-005	0.997205	1/x	0.029	25
Tre	16.90237	y = 0.560146 x - 2.640968E-004	0.993141	1/x	0.003	25
Raffinose	22.801	y = 0.130197 x + 6.717587E-006	0.999059	1/x	0.021	25

1) Index is the substance name
2) Equation is the linear equation
3) r is the correlation coefficient
4) Weighting is the weight
5) LLOQ (ng/mL) and ULOQ (ng/mL) are the lower limit of quantification and upper limit of quantification, respectively. Accurate quantification is possible within these limits.

Table S3 Organic acid concentration (ng/g)

Index	Group	Mean	SD	SE
L-malic-acid	Q60	10803423	206786	119388
L-malic-acid	S120	8138948	94833	54752
L-malic-acid	S60	7719514	129493	74763
L-malic-acid	Q120	7479561	48988	28283
cryptochlorogenic-acid	S60	265074	10132	5849.9
cryptochlorogenic-acid	S120	150034	4371.2	2523.7
maslinic-acid	S120	63614	32097	18531
oxoglutaric-acid	Q60	54360	1505.2	869.05
maslinic-acid	Q60	39447	14846	8571.2
cis-aconitic-acid	Q60	37408	1212.8	700.24
4-aminobutyric-acid	S60	35795	365.49	211.02
maslinic-acid	S60	29628	9538.3	5506.9
cis-aconitic-acid	S60	27468	1317.9	760.89
maslinic-acid	Q120	27194	4380.4	2529.1
cis-aconitic-acid	S120	26242	827.24	477.61
cryptochlorogenic-acid	Q60	26151	901.89	520.71
succinic-acid	Q60	24833	680.18	392.7
succinic-acid	Q120	22970	905.89	523.01
4-aminobutyric-acid	Q60	21618	185.25	106.96
tartaric-acid	S120	17823	262.4	151.49
tartaric-acid	S60	16842	321.99	185.9
tartaric-acid	Q60	16325	212.02	122.41
cis-aconitic-acid	Q120	14917	131.34	75.827
succinic-acid	S60	14636	916.26	529
oxoglutaric-acid	S120	12792	547.28	315.97
tartaric-acid	Q120	11309	243.04	140.32
succinic-acid	S120	11232	598.35	345.46
pantothenic-acid	Q60	10497	237.06	136.87
shikimic-acid	Q60	10229	1033.5	596.69
pyruvic-acid	Q60	10137	397.79	229.67
pantothenic-acid	S120	9957.4	328.13	189.45
oxoglutaric-acid	Q120	9828.1	107.56	62.098
shikimic-acid	S60	9605.4	409.99	236.71
oxoglutaric-acid	S60	8932.7	726.68	419.55
cryptochlorogenic-acid	Q120	8249.6	540.74	312.2
4-aminobutyric-acid	S120	6566.1	277.08	159.97
pantothenic-acid	Q120	6444.7	414.91	239.55
pyruvic-acid	Q120	5747.7	269.47	155.58
pyruvic-acid	S120	5346.5	374.5	216.22
pantothenic-acid	S60	5270.6	293.38	169.38
shikimic-acid	S120	4452.9	115.89	66.911
pyroglutamic-acid	S60	4169.3	142.52	82.286
pyruvic-acid	S60	3954.2	92.952	53.666
4-aminobutyric-acid	Q120	3781.8	78.116	45.1
2-methylsuccinic-acid	S60	3559.9	180.68	104.32

neochlorogenic-acid	S120	3257.1	218.3	126.04
2-methylsuccinic-acid	Q60	2660.5	70.883	40.924
2-methylsuccinic-acid	Q120	2627.8	140.66	81.209
pyroglutamic-acid	Q60	2404.4	135.79	78.4
2-methylsuccinic-acid	S120	2121.1	129.44	74.735
neochlorogenic-acid	S60	1906.6	74.174	42.824
pyroglutamic-acid	S120	1742.5	44.49	25.686
shikimic-acid	Q120	1492.8	211.17	121.92
3-hydroxymethylglutaric-acid	S120	1489.7	55.118	31.822
3-hydroxymethylglutaric-acid	Q120	1385	84.043	48.522
2-hydroxy-2-methylbutyric-acid	S120	1164.5	80.464	46.456
pyroglutamic-acid	Q120	1066.8	48.658	28.093
glutaric-acid	S60	996.13	235.88	136.19
glutaric-acid	Q60	848.07	112.93	65.202
taurine	S60	650.88	24.902	14.377
3-hydroxymethylglutaric-acid	S60	545.71	18.839	10.877
3-hydroxyisovaleric-acid	S60	521.46	32.721	18.892
ferulic-acid	S60	468.71	15.078	8.7055
3-hydroxyisovaleric-acid	S120	406.88	24.49	14.14
4-coumaric-acid	S60	363.06	23.129	13.354
3-hydroxymethylglutaric-acid	Q60	336.55	25.661	14.815
4-coumaric-acid	S120	245.05	11.849	6.8412
4-coumaric-acid	Q120	237.6	20.083	11.595
2-hydroxy-2-methylbutyric-acid	S60	236.19	16.436	9.4893
4-coumaric-acid	Q60	207.31	14.956	8.6347
adipic-acid	S60	187.77	70.027	40.43
adipic-acid	Q120	144.54	44.408	25.639
adipic-acid	Q60	134.71	24.781	14.307
2-hydroxy-2-methylbutyric-acid	Q120	128.33	15.891	9.1746
adipic-acid	S120	120.57	11.747	6.7823
3-hydroxyisovaleric-acid	Q120	102.16	13.821	7.9795
ferulic-acid	Q120	101.78	6.7816	3.9154
3-phenyllactic-acid	Q60	86.426	4.4305	2.5579
hydroxyphenyllactic-acid	S120	80.365	8.1026	4.678
ferulic-acid	S120	75.396	3.8078	2.1985
caffeic-acid	Q60	74.794	5.8015	3.3495
caffeic-acid	Q120	59.618	9.7862	5.65
4-hydroxybenzoic-acid	Q120	50.813	2.9659	1.7124
3-hydroxyisovaleric-acid	Q60	39.901	11.247	6.4936
cinnamic-acid	Q120	33.562	4.2109	2.4312
hydroxyphenyllactic-acid	Q60	28.925	1.9215	1.1094
3-Hydroxyphenyl-hydracrylic-acid	S120	16.16	2.5273	1.4592
3-Hydroxyphenyl-hydracrylic-acid	Q120	9.428	0.44516	0.25701
4-hydroxybenzoic-acid	S120	8.6351	1.6779	0.96876
kynurenic-acid	S120	8.3891	0.53996	0.31174
3-Hydroxyphenyl-hydracrylic-acid	Q60	7.9436	1.3401	0.77371
kynurenic-acid	S60	7.1552	0.50709	0.29277

5-hydroxyindoleacetic-acid	S60	6.319	0.9529	0.55015
kynurenic-acid	Q120	5.3515	0.57517	0.33207
phenaceturic-acid	S120	5.1441	0.98101	0.56638
5-hydroxyindoleacetic-acid	Q60	5.0158	0.64683	0.37345
gallic-acid	S60	4.7009	0.7637	0.44092
phenaceturic-acid	Q120	4.4996	0.72669	0.41956
4-hydroxyhippuric-acid	S120	4.296	1.2742	0.73564
4-hydroxyhippuric-acid	S60	3.6257	0.32739	0.18902
gallic-acid	Q60	2.4575	0.33189	0.19162
gallic-acid	Q120	1.9171	0.29784	0.17196
4-hydroxyhippuric-acid	Q120	1.0137	0.10084	0.058222
4-hydroxyhippuric-acid	Q60	0.84376	0.27088	0.15639
indole-3-acetic-acid	S60	0.74978	0.098811	0.057049
indole-3-acetic-acid	Q60	0.70803	0.049639	0.028659
indole-3-acetic-acid	Q120	0.5509	0.021089	0.012176
2-hydroxy-2-methylbutyric-acid	Q60	0	0	0
3-Hydroxyphenyl-hydracrylic-acid	S60	0	0	0
3-phenyllactic-acid	Q120	0	0	0
3-phenyllactic-acid	S120	0	0	0
3-phenyllactic-acid	S60	0	0	0
4-hydroxybenzoic-acid	Q60	0	0	0
4-hydroxybenzoic-acid	S60	0	0	0
5-hydroxyindoleacetic-acid	Q120	0	0	0
5-hydroxyindoleacetic-acid	S120	0	0	0
caffeic-acid	S120	0	0	0
caffeic-acid	S60	0	0	0
cinnamic-acid	S120	0	0	0
cinnamic-acid	Q60	0	0	0
cinnamic-acid	S60	0	0	0
ferulic-acid	Q60	0	0	0
gallic-acid	S120	0	0	0
glutaric-acid	Q120	0	0	0
glutaric-acid	S120	0	0	0
hydroxyphenyllactic-acid	Q120	0	0	0
hydroxyphenyllactic-acid	S60	0	0	0
indole-3-acetic-acid	S120	0	0	0
kynurenic-acid	Q60	0	0	0
neochlorogenic-acid	Q120	0	0	0
neochlorogenic-acid	Q60	0	0	0
phenaceturic-acid	Q60	0	0	0
phenaceturic-acid	S60	0	0	0
taurine	Q120	0	0	0
taurine	S120	0	0	0
taurine	Q60	0	0	0

Table S4 Sugar concentration (mg/g)

Index	Group	Mean	SD	SE
Fru	Q120	160.44	4.3823	2.5301
Fru	S60	147.88	4.0349	2.3295
Suc	Q120	138.35	4.2983	2.4816
Suc	S120	137.07	0.192	0.11085
Fru	S120	133.85	1.6712	0.96489
Glu	Q120	112.47	2.5378	1.4652
Glu	S60	111.57	5.629	3.2499
Fru	Q60	109.98	5.6405	3.2565
Glu	S120	87.738	0.62668	0.36181
Suc	S60	84.994	3.8964	2.2496
Glu	Q60	80.621	4.3723	2.5244
Sorbitol	S120	70.623	0.68522	0.39561
Suc	Q60	65.42	3.5454	2.0469
Sorbitol	Q120	43.579	1.3779	0.79556
Sorbitol	Q60	29.219	1.6959	0.97914
Sorbitol	S60	28.58	0.17267	0.099693
Xylose	Q120	5.3507	0.31231	0.18031
Xylose	S120	4.0108	0.041221	0.023799
Gal	S120	1.2552	0.012694	0.007329
Inositol	S60	1.163	0.027358	0.015795
Inositol	S120	1.1411	0.0152	0.008776
Xylose	S60	1.1328	0.009508	0.00549
Inositol	Q120	1.0233	0.065649	0.037902
Xylose	Q60	0.81731	0.065506	0.03782
Gal	Q120	0.54745	0.040128	0.023168
Inositol	Q60	0.54688	0.040807	0.02356
Mal	S120	0.54225	0.006051	0.003494
Glucuronic-A	S120	0.51227	0.008118	0.004687
Glucuronic-A	Q120	0.32093	0.011252	0.006497
Gal	S60	0.31051	0.009139	0.005276
Man	Q120	0.23734	0.016427	0.009484
Glucuronic-A	S60	0.23151	0.002998	0.001731
Man	S60	0.2278	0.00238	0.001374
Glucuronic-A	Q60	0.22409	0.01348	0.007783
Gal	Q60	0.18759	0.010605	0.006123
Arabinitol	Q120	0.17997	0.003297	0.001904
Xylitol	S120	0.17498	0.001583	0.000914
Arabinitol	Q60	0.16696	0.006412	0.003702
Arabinitol	S60	0.15968	0.000953	0.00055
Man	Q60	0.15117	0.010084	0.005822
Lev	Q120	0.15102	0.010078	0.005819
Lev	S120	0.13053	0.003954	0.002283
Man	S120	0.11683	0.004374	0.002526
Arabinitol	S120	0.10796	0.00087	0.000502
Ribose	S120	0.10398	0.000263	0.000152

Ribose	Q120	0.094334	0.000728	0.00042
D-Ara	S120	0.087966	0.002595	0.001498
Lev	Q60	0.072161	0.005555	0.003207
Xylitol	Q120	0.070991	0.002936	0.001695
Rha	S120	0.069819	0.000726	0.000419
D-Ara	Q120	0.062376	0.002075	0.001198
Gal-A	S120	0.061292	0.001648	0.000952
Man-6-pho	S60	0.058522	0.006216	0.003589
Rha	Q120	0.058155	0.002961	0.00171
Fuc	S120	0.057838	0.00125	0.000722
Lev	S60	0.052226	0.001563	0.000902
Ribose	S60	0.049502	0.000729	0.000421
Rha	Q60	0.045088	0.002945	0.0017
Mal	Q120	0.042116	0.003044	0.001757
Ribose-5-pho-Ba	S120	0.041247	0.001384	0.000799
Man-6-pho	Q60	0.035169	0.002856	0.001649
Rha	S60	0.034129	0.000104	5.98E-05
Ribose-5-pho-Ba	Q120	0.032412	0.000662	0.000382
Raffinose	S60	0.031457	0.003392	0.001958
D-Ara	S60	0.031416	0.00198	0.001143
Gal-A	Q120	0.031391	0.003631	0.002096
Ribose-5-pho-Ba	S60	0.03119	0.003369	0.001945
Fuc	S60	0.03107	0.001206	0.000696
Fuc	Q120	0.030387	0.000785	0.000453
Ribose	Q60	0.030097	0.000822	0.000474
Ribose-5-pho-Ba	Q60	0.028325	0.000966	0.000558
Man-6-pho	S120	0.027759	0.002784	0.001607
Mal	S60	0.027245	0.001801	0.00104
D-Ara	Q60	0.026148	0.00139	0.000803
Xylitol	Q60	0.025291	0.001759	0.001015
Xylitol	S60	0.025057	0.000159	9.19E-05
Fuc	Q60	0.022828	0.001505	0.000869
2-Ace-2-Deo-D-Glucosamine	Q120	0.022585	0.000916	0.000529
2-Ace-2-Deo-D-Glucosamine	Q60	0.021241	0.001064	0.000614
Man-6-pho	Q120	0.02032	0.002191	0.001265
Raffinose	Q60	0.019342	0.001469	0.000848
Raffinose	Q120	0.018913	0.000442	0.000255
Tre	S120	0.014184	0.000137	7.89E-05
2-Ace-2-Deo-D-Glucosamine	S60	0.013945	0.000879	0.000508
Ribono-1-4-lactone	S60	0.013444	0.000688	0.000397
Ribono-1-4-lactone	Q120	0.013172	0.000437	0.000252
2-Ace-2-Deo-D-Glucosamine	S120	0.013118	0.000131	7.57E-05
Raffinose	S120	0.012108	0.000862	0.000498
Tre	S60	0.011075	0.000496	0.000286
Tre	Q120	0.010551	0.000336	0.000194
Ribono-1-4-lactone	S120	0.010521	6.63E-05	3.83E-05
Ribono-1-4-lactone	Q60	0.009866	0.000953	0.00055

Tre	Q60	0.008693	0.000378	0.000218
Deo	Q120	0.007869	0.000239	0.000138
Gal-A	S60	0.006934	0.00013	7.49E-05
Deo	Q60	0.006845	0.000305	0.000176
Deo	S120	0.006412	1.98E-05	1.15E-05
Deo	S60	0.006087	0.000217	0.000125
Gal-A	Q60	0.004713	0.000228	0.000131
Mal	Q60	0.004298	0.000104	6.02E-05
Xylulose	Q120	0.004209	0.000376	0.000217
Phe	S120	0.001102	3.92E-05	2.26E-05
Phe	Q120	0.000994	2.69E-05	1.55E-05
Phe	Q60	0.000735	7.29E-05	4.21E-05
Phe	S60	0.000656	2.58E-05	1.49E-05
Xylulose	S120	0	0	0
Xylulose	Q60	0	0	0
Xylulose	S60	0	0	0

Table S5 Canonical correlation analysis of differential metabolites and differential genes related to organic acids

Contrast	KEGG_map	Description	U1V1	U2V2	U3V3	U4V4	U5V5
Q60-S60	ko01110	Biosynthesis of secondary metabolites	0.9998	0.999	0.971059	--	--
	ko00040	Pentose and glucuronate interconversions	0.9956	0.9527	--	--	--
	ko00650	Butanoate metabolism	0.9835	0.8913	--	--	--
	ko00053	Ascorbate and aldarate metabolism	0.992	0.9186	--	--	--
	ko00430	Taurine and hypotaurine metabolism	0.9995	0.9981	0.897044	--	--
	ko01240	Biosynthesis of cofactors	0.9962	0.9513	--	--	--
	ko00250	Alanine, aspartate and glutamate metabolism	0.9913	0.9154	--	--	--
	ko01210	2-Oxocarboxylic acid metabolism	0.7913	0.1811	--	--	--
	ko00630	Glyoxylate and dicarboxylate metabolism	0.9934	0.9299	--	--	--
	ko00020	Citrate cycle (TCA cycle)	0.8136	0.1988	--	--	--
	ko01200	Carbon metabolism	0.998	0.973	--	--	--
	ko01230	Biosynthesis of amino acids	0.9346	0.359	--	--	--
	ko00660	C5-Branched dibasic acid metabolism	0.8939	0.4095	--	--	--
	ko00785	Lipoic acid metabolism	0.8135	0.1986	--	--	--
Q120-S120	ko01110	Biosynthesis of secondary metabolites	0.9991	0.9983	0.981838	--	--
	ko01240	Biosynthesis of cofactors	0.9906	0.9694	--	--	--
	ko00999	Biosynthesis of various plant secondary metabolites	0.9798	0.9542	--	--	--
Q60-Q120	ko01110	Biosynthesis of secondary metabolites	0.8738	0.7777	0.43104	0.08693	0.059269
	ko01240	Biosynthesis of cofactors	0.8327	0.6219	0.312142	--	--
	ko00630	Glyoxylate and dicarboxylate metabolism	0.7967	0.4098	--	--	--
	ko00660	C5-Branched dibasic acid metabolism	0.8	0.412	--	--	--
	ko00650	Butanoate metabolism	0.9743	0.887	--	--	--
	ko00020	Citrate cycle (TCA cycle)	0.7953	0.4095	--	--	--
	ko01210	2-Oxocarboxylic acid metabolism	0.8012	0.4146	--	--	--
	ko00250	Alanine, aspartate and glutamate metabolism	0.9885	0.9626	--	--	--
	ko01230	Biosynthesis of amino acids	0.7821	0.546	--	--	--
	ko00310	Lysine degradation	0.7503	0.5547	--	--	--
S60-S120	ko01110	Biosynthesis of secondary metabolites	0.817	0.6762	0.333347	--	--
	ko00999	Biosynthesis of various plant secondary metabolites	0.9855	0.9507	--	--	--
	ko01240	Biosynthesis of cofactors	0.8073	0.338	--	--	--

1) KEGG_map: KEGG database pathway number
2) Description: Pathway description
3) UnVn: The canonical correlation coefficient for the nth pair of canonical variables. The closer its absolute value is to 1, the stronger the correlation between the canonical variables.

Table S6 Canonical correlation analysis of differential metabolites and differential genes related to sugar

Contrast	KEGG_map	Description	U1V1	U2V2	U3V3	U4V4	U5V5
Q60-S60	ko01110	Biosynthesis of secondary metabolites	0.9229	0.831	--	--	--
	ko02010	ABC transporters	0.7542	0.5694	--	--	--
Q120-S120	ko00052	Galactose metabolism	0.9927	0.9712	--	--	--
	ko00040	Pentose and glucuronate interconversions	0.9844	0.9783	--	--	--
	ko02010	ABC transporters	0.9997	0.9988	0.978935	--	--
Q60-Q120	ko01110	Biosynthesis of secondary metabolites	0.784	0.5367	--	--	--
	ko00040	Pentose and glucuronate interconversions	0.8867	0.7534	0.072446	0.042806	0.028548
	ko00052	Galactose metabolism	0.7972	0.4188	--	--	--
	ko00520	Amino sugar and nucleotide sugar metabolism	0.8544	0.4765	0.047858	--	--
	ko00053	Ascorbate and aldarate metabolism	0.8159	0.0893	--	--	--
	ko01250	Biosynthesis of nucleotide sugars	0.8159	0.0893	--	--	--
	ko02010	ABC transporters	0.9894	0.9472	0.906142	0.503823	0.394623
	ko00500	Starch and sucrose metabolism	0.784	0.5367	--	--	--
S60-S120	ko00040	Pentose and glucuronate interconversions	0.9666	0.7914	0.121319	0.071089	--
	ko00051	Fructose and mannose metabolism	0.9997	0.9933	--	--	--
	ko00052	Galactose metabolism	0.9998	0.9985	0.838364	--	--
	ko00520	Amino sugar and nucleotide sugar metabolism	0.8544	0.4765	0.047845	--	--
	ko01250	Biosynthesis of nucleotide sugars	0.8159	0.0893	--	--	--
	ko02010	ABC transporters	0.9893	0.954	0.933452	0.503622	0.413748
	ko00053	Ascorbate and aldarate metabolism	0.8065	0.0814	--	--	--

1) KEGG_map: KEGG database pathway number

2) Description: Pathway description

3) UnVn: The canonical correlation coefficient for the nth pair of canonical variables. The closer its absolute value is to 1, the stronger the correlation between the canonical variables.

Table S7 Screening of key components for acid-sugar conversion analysis

Components (μg/g)	**S60-1**	**S60-2**	**S60-3**	**Q60-1**	**Q60-2**	**Q60-3**	**S120-1**	**S120-2**	**S120-3**	**Q120-1**	**Q120-2**	**Q120-3**	Concentration advantage	Differential components	Pathway dominance
L-malic acid	7868.99	7641.56	7647.99	10573.60	10862.23	10974.44	8111.58	8244.46	8060.81	7534.89	7441.70	7462.09	+	\	\
cryptochlorogenic-acid	276.50	261.56	257.17	26.94	26.34	25.17	151.53	153.46	145.11	8.73	8.35	7.67	+	+	\
maslinic-acid	30.93	38.45	19.50	53.82	40.34	24.17	60.53	33.17	97.14	32.25	24.77	24.56	+	+	\
cis-aconitic-acid	28.94	26.40	27.06	37.43	38.61	36.18	26.76	26.68	25.29	14.94	14.78	15.04	+	+	\
oxoglutaric-acid	8.51	9.77	8.52	56.04	53.92	53.12	12.55	13.42	12.40	9.90	9.89	9.70	+	+	+
succinic-acid	15.69	14.08	14.14	25.23	25.22	24.05	11.92	10.81	10.97	23.71	23.24	21.96	+	+	\
4-aminobutyric-acid	36.22	35.58	35.59	21.41	21.77	21.68	6.26	6.64	6.80	3.76	3.72	3.87	+	+	\
tartaric-acid	16.91	16.49	17.13	16.09	16.40	16.49	17.69	18.13	17.65	11.58	11.22	11.12	+	\	\
pyruvic acid	3.88	4.06	3.93	9.69	10.46	10.26	5.11	5.78	5.15	5.84	5.96	5.45	\	+	+
4-hydroxybenzoic acid	0.00	0.00	0.00	0.00	0.00	0.00	0.01	0.01	0.01	0.05	0.05	0.05	\	+	\
3-hydroxyisovaleric acid	0.50	0.50	0.56	0.04	0.05	0.03	0.42	0.42	0.38	0.10	0.12	0.09	\	+	\
shikimic acid	10.07	9.28	9.46	11.08	10.53	9.08	4.53	4.51	4.32	1.73	1.42	1.32	\	+	\
4-hydroxyhippuric acid	0.00	0.00	0.00	0.00	0.00	0.00	0.01	0.00	0.00	0.00	0.00	0.00	\	+	\
2-hydroxy-2-methylbutyric acid	0.25	0.22	0.23	0.00	0.00	0.00	1.25	1.10	1.15	0.13	0.14	0.11	\	+	\
3-hydroxymethylglutaric acid	0.56	0.55	0.53	0.36	0.34	0.31	1.51	1.43	1.54	1.47	1.39	1.30	\	+	\
pyroglutamic acid	4.33	4.08	4.09	2.43	2.52	2.26	1.79	1.72	1.71	1.12	1.05	1.03	\	+	\
ferulic acid	0.48	0.47	0.45	0.00	0.00	0.00	0.08	0.08	0.07	0.11	0.11	0.09	\	+	\
D-fructose	150.69	149.69	143.25	110.74	104.00	115.20	132.48	133.37	135.71	156.66	159.41	165.25	+	\	\
sucrose	86.70	87.75	80.54	66.99	61.36	67.91	137.27	136.89	137.04	138.07	134.20	142.79	+	+	+
glucose	114.16	115.44	105.11	81.36	75.93	84.58	87.44	87.32	88.46	109.86	112.62	114.93	+	\	\
maltose	0.03	0.03	0.03	0.00	0.00	0.00	0.54	0.54	0.55	0.04	0.04	0.05	\	+	+
inositol	1.17	1.13	1.19	0.56	0.50	0.58	1.12	1.14	1.15	0.95	1.06	1.07	\	+	+
D-galactose	0.32	0.30	0.31	0.19	0.18	0.20	1.24	1.27	1.26	0.50	0.56	0.58	\	+	+
D-mannose	0.23	0.23	0.23	0.15	0.14	0.16	0.11	0.12	0.12	0.22	0.24	0.25	\	+	+
xylitol	0.02	0.03	0.03	0.03	0.02	0.03	0.17	0.18	0.18	0.07	0.07	0.07	\	+	+
D-galacturonic acid	0.01	0.01	0.01	0.00	0.00	0.00	0.06	0.06	0.06	0.03	0.03	0.03	\	+	+

D-xylose	1.14	1.13	1.12	0.84	0.74	0.87	3.96	4.04	4.03	5.00	5.47	5.59	\	+	\
D-ribose	0.05	0.05	0.05	0.03	0.03	0.03	0.10	0.10	0.10	0.09	0.09	0.10	\	+	+
D-arabinose	0.03	0.03	0.03	0.03	0.02	0.03	0.09	0.09	0.09	0.06	0.06	0.06	\	+	+
levoglucosan	0.05	0.05	0.05	0.07	0.07	0.08	0.13	0.13	0.13	0.14	0.16	0.16	\	+	\
raffinose	0.03	0.03	0.03	0.02	0.02	0.02	0.01	0.01	0.01	0.02	0.02	0.02	\	+	+
D-Mannose-6P-Na	0.06	0.06	0.05	0.04	0.03	0.04	0.02	0.03	0.03	0.02	0.02	0.02	\	+	\
D-Sorbitol	28.72	28.39	28.63	29.82	27.31	30.54	69.98	70.54	71.34	41.99	44.27	44.47	\	+	+
L-rhamnose	0.03	0.03	0.03	0.05	0.04	0.05	0.07	0.07	0.07	0.05	0.06	0.06	\	+	+
D-Xylulose	0.000	0.000	0.000	0.000	0.000	0.000	0.000	0.000	0.000	0.004	0.005	0.004	\	\	+

Information on concentration advantage, different components, and pathway dominance was obtained from graphs 1, 2, 4, and 3, 5, respectively. "+" indicates that the component possesses the corresponding attribute
